# Direct incorporation of mesenchymal stem cells into a Nanofiber scaffold – *in vitro* and *in vivo* analysis

**DOI:** 10.1038/s41598-020-66281-6

**Published:** 2020-06-12

**Authors:** Karl F. Schüttler, Michael W. Bauhofer, Vanessa Ketter, Katja Giese, Daphne A. Eschbach, Mesut Yenigün, Susanne Fuchs-Winkelmann, Jürgen R. J. Paletta

**Affiliations:** 1 Center for Orthopedics and Trauma Surgery, University Hospital Giessen and Marburg, Location Marburg, Marburg, Germany; 2 Department of Neurology, University Hospital Giessen and Marburg, Location Giessen, Giessen, Germany

**Keywords:** Tissue engineering, Stem-cell research

## Abstract

Bony defects are a common problem in musculoskeletal surgery. Replacement with autologous bone grafts is limited by availability of transplant material. Sterilized cancellous bone, while being osteoconductive, has limited osteoinductivity. Nanofiber scaffolds are currently used for several purposes due to their capability of imitating the extracellular matrix. Furthermore, they allow modification to provide functional properties. Previously we showed that electrospun nanofiber scaffolds can be used for bone tissue regeneration. While aiming to use the osteoinductive capacities of collagen type-I nanofibers we saw reduced scaffold pore sizes that limited cellular migration and thus colonization of the scaffolds. Aim of the present study was the incorporation of mesenchymal stem cells into the electrospinning process of a nanofiber scaffold to produce cell-seeded nanofiber scaffolds for bone replacement. After construction of a suitable spinning apparatus for simultaneous electrospinning and spraying with independently controllable spinning and spraying devices and extensive optimization of the spinning process, *in vitro* and *in vivo* evaluation of the resulting scaffolds was conducted. Stem cells isolated from rat femora were incorporated into PLLA (poly-l-lactide acid) and PLLA-collagen type-I nanofiber scaffolds (PLLA Col I Blend) via simultaneous electrospinning and –spraying. Metabolic activity, proliferation and osteoblastic differentiation were assessed *in vitro*. For *in vivo* evaluation scaffolds were implanted into critical size defects of the rat scull. After 4 weeks, animals were sacrificed and bone healing was analyzed using CT-scans, histological, immunhistochemical and fluorescence evaluation. Successful integration of mesenchymal stem cells into the scaffolds was achieved by iteration of spinning and spraying conditions regarding polymer solvent, spinning distance, the use of a liquid counter-electrode, electrode voltage and spinning duration. *In vivo* formation of bone tissue was achieved. Using a PLLA scaffold, comparable results for the cell-free and cell-seeded scaffolds were found, while the cell-seeded PLLA-collagen scaffolds showed significantly better bone formation when compared to the cell-free PLLA-collagen scaffolds. These results provide support for the future use of cell-seeded nanofiber scaffolds for large bony defects.

## Introduction

Bony defects are a common problem in musculoskeletal surgery e.g. after tumor resection, infection, revision arthroplasty or trauma. In general, studies have shown that the physiological process of fracture healing is disturbed in 5–30% which results in delayed or incomplete recovery, depending on the treatment^1^. To treat such defects, adequate bone replacement is needed. As this affects approximately 500,000 surgeries in Europe and approximately 2.2 million surgeries per year worldwide, bone is the second most transplanted tissue^2,3^. Although replacement with autologous bone is the “golden standard”, this procedure is limited by availability of transplant material and sterilized cancellous bone, while being osteoconductive, has limited osteoinductivity^3–6^. As a consequence, multiple artificial scaffolds for bone replacement on the basis of ceramics, polymer foams, membranes, gels or composite materials are currently under investigation^7–16^.

In this context nanofiber-based scaffolds are of interest due to their ability to mimic the extracellular matrix to some extent^17–23^. The possibility to control the structural, mechanical and biophysical properties of the scaffold by choosing from different suitable polymers, co-polymers and changing spinning-conditions is another advantage of nanofiber scaffolds^24–28^.

Among the different polymers suitable for electro spinning, poly-(l-lactic acid) (PLLA) is a polymer which offers the advantage of being approved by the Food and Drug Administration (FDA) in addition to PLLA products or devices being already used in the field of bone surgery^29–35^.

In the context of tissue engineering, PLLA-nanofibers can promote growth and differentiation of human mesenchymal stem cells^36,37^. Furthermore, the differentiability of mesenchymal stem cells to osteoblasts could also be demonstrated in principle^38,39^. However, this process appears to be slowed when using PLLA nanofibers electroplated from dichloromethane. Thus, own studies show an initially reduced expression of genes involved in osteoblastic differentiation^37^.

In this context it is obvious to improve the osteoinductive capacities of a PLLA based nanofiber scaffold. In many tissues, collagen is the leading structural element of the extracellular matrix^40^. Using HFIP (1,1,3,3-hexafluoro-2-propanol), collagen can be spun into nanofiber mats. The fiber diameter varies as a function of concentration, voltage, electrode spacing and flow rate between 100 and 730 nm^41^.

Regarding the growth of stem cells, our own work has shown that collagen nanofibers (2D-mats) achieve higher cell densities than PLLA nanofibers or glass surfaces^37^. In addition, there is an osteoinductive effect which is independent of whether the cells were cultured under growth or osteoinductive conditions^36^. Thus, the functionalization of PLLA by means of including collagen type I seems to be a tried-and-tested method of increasing the osteoinductive potential of PLLA nanofibers. However, these desired biological effects of these functionalized PLLA-collagen type I scaffolds (PLLA Col I Blend) are accompanied by an undesired “side-effect”. The pore size of these functionalized PLLA-collagen scaffolds dropped to 0.5–2 μm^42^. *In vivo* this resulted in a limited cellular migration and thus colonization of the scaffolds^42,43^. The lower limit for successful scaffold colonization according to Szentivanyi seems a pore size of approximate 5 μm^44^. As a consequence, no increase in bone formation *in-vivo*, despite maintenance of the scaffold’s osteoinductivity, could be achieved in our previous works^42^. In order to overcome this disadvantage a direct incorporation of living cells into nanofiber scaffolds during electrospinning could be a promising approach for producing cell-containing scaffolds. This approach of direct cellular incorporation could provide scaffolds with high initial cell density, which was shown to have an important effect on biomechanical stability of tissue engineered constructs^45,46^. A critical limitation within such a setting is the mandatory supply with nutrients for these cell-seeded scaffolds. Based on theoretical models approximately 280000 cells/cm^3^ can survive without central necrosis within a scaffold of 1 cm thickness^47^.

**Figure 1:**
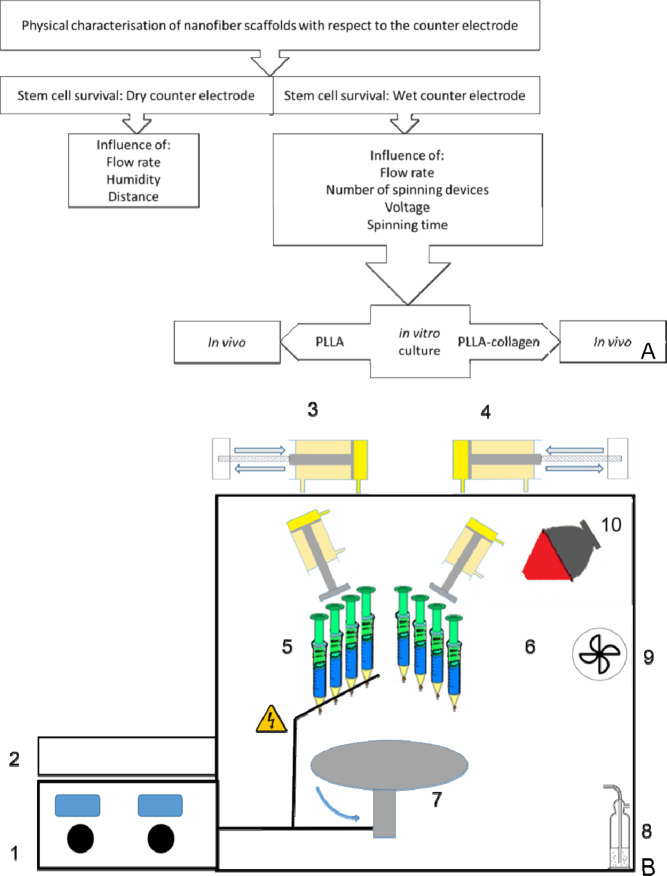
Procedure and spinning apparatus in the development of cell seeded scaffolds. Flow chart of the optimization process (**A**) and schematic of the spinning apparatus (**B**) 1. Voltage source varying from 0–30 kV (2 independent); 2. Motor control for two hydraulic pumps; 3.&4. Hydraulic pumps with stepper motor and threaded rod; 5.&6. 2 independent spinning heads consisting of 4 syringes each; 7. Rotating counter electrode; 8. Humidification unit & air supply; 9. Air extraction; 10. Heat regulation lamp.

Based on a combination of electro spraying of cells with electro spinning of nanofiber scaffold a process which was described for cardiovascular tissue engineering^48^, osteosarcoma cell line^49^, or MSC^50^ a spinning apparatus was constructed allowing for direct incorporation of cells via electro spraying into the electrospinning process (see Material and Methods; Fig. 1).

The aim of the present study was thus the production of different cell-seeded nanofiber scaffolds for bone replacement, the optimization of the simultaneous spinning- and spraying-process and the evaluation of the resulting scaffolds *in vitro* and *in vivo* in a critical size bone defect model (Fig. 1).

## Results

### Optimization

**Figure 2:**
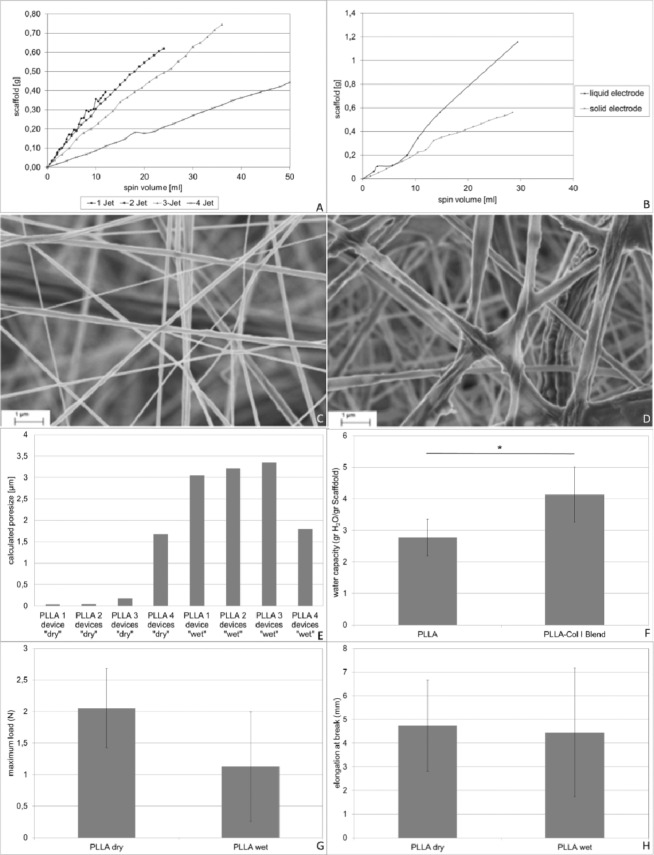
Physical characterization of PLLA Nanofiber scaffolds obtained by a multi-jet electrospinning. Influence of multi-jet electrospinning (**A**) and type of counter electrode (**B**) on scaffold mass deposition. Nanofibers obtained by a dry (**C**) or wet (**D**) counter electrode. Influence of multi-jet electrospinning and type of counter electrode on calculated pore size (**E**). Mechanic stability in dependence of the counter electrode (**G,H**) and water capacity of the scaffolds (**F**).

The optimization process is summarized in Fig. 2 (Fig. 2). In order to analyze the influence of multi-jet electrospinning we determined the increase of scaffold mass in dependence of 1 to 4 spraying devices. PLLA was dissolved in Dichloromethane-Methanol (DCM/MeOH) and electro spun from 1 to 4 spinning devices. Spinning voltage was adjusted to 25 kV and the spinning distance was set to 6 cm. An aluminum counter electrode of 100 cm^2^ was used to collect the fibers (−5 kV counter voltage). As shown in Fig. 2 (Fig. 2A) the efficiency in fiber deposition decreased with the number of spraying devices after using more than two devices. Changing from two to three devices the yield decreased from 100% to 30% of the theoretically achievable scaffold mass with a distinct decrease in the 4-device system. The use of 1 or 2 devices showed only minimal differences in the scaffold deposition (103% vs 93% of the theoretical achievable scaffold mass) (Fig. 2A).

Fiber diameter showed no significant differences when the number of spinning devices was increased up to 2 devices (p = 0.259). A mean fiber diameter of 180 nm and a mean porosity of 81% were found within the dry counter electrode system using two spinning devices (Fig. 2C).

Due to the improved cell survival obtained when using a liquid counter electrode, we analyzed the scaffold formation on liquid counter electrodes. Comparing the scaffold formation on a dry aluminum counter electrode with a liquid counter electrode filled with DMEM cell culture medium using 3 spinning devices we found a higher scaffold mass representing a higher polymer retrieval rate of 90 ± 14% by using the liquid counter electrode compared to the 30% using a dry counter electrode (Fig. 1B).

The use of a liquid counter electrode resulted in a significantly increased mean fiber diameter in the 1, 2 and 3 device setting when compared to the dry counter electrode (557 nm vs. 180 nm; p < 0.001; Fig. 2D). No significant difference was found in the 4 device set-up between wet and dry counter electrode. Mean scaffold porosity increased slightly up to 83%. Focusing on the 1, 2 and 3 device setting the, calculated pore size increased from below 0.5 µm to above 3 µm in the liquid counter electrode set-up (Fig. 2E). Only the 4 device setting seems to represent an exception. With respect to mechanical properties of the scaffolds, the introduction of a wet counter-electrode led to non-significant changes regarding maximum load (p = 0.12) and elongation at brake (p = 0.86) (Fig. 2G,H). When comparing PLLA with PLLA-Col I Blend scaffolds, significant differences were found regarding water capacity, with almost double the amount of water uptake per scaffold (w/w) in the PLLA-Col I Blend scaffolds (Fig. 2F, p = 0.037).

Based on earlier findings, showing that immortalized osteoblastic cell lines could be incorporated into nanofiber scaffolds by a coaxial process combining electro spinning and electro spraying^49^, the aim was to incorporate bone marrow derived pluripotent mesenchymal cells with a CD90 and CD105 positive phenotype into the nanofiber scaffolds.

In order to characterize the influence of different parameter on cell survival after incorporation into a PLLA nanofiber scaffold we analyzed the influence of Humidity (30–80%), spinning distance (6 vs. 12 cm), flow rate (2–12 ml/h), voltage (0–30 kV), counter electrode (dry bare aluminum vs. a petri disc containing DMEM (Dulbecco’s Modified Eagle Medium) as a liquid counter electrode), solvent (DCM vs. DCM/MeOH vs. HFIP) as well as polymer (PLLA vs. PLLA Col I Blend).

Cells were suspended in DMEM and electro sprayed from one to four devices with a flow rate of 2, 6 and 12 ml/h while the voltage was varied between 0–30 kV. Scaffolds were built from 2 devices with a flow rate of 0,5 ml/h at a voltage of 25 kV using either DCM or DCM/MeOH as solvent when spinning PLLA scaffolds or HFIP as solvent when spinning PLLA Col I Blend scaffolds. Obtained scaffolds were analyzed by FDA/EtBr staining directly after scaffold production or by MTT assay after a culture period of 24 h.

**Figure 3:**
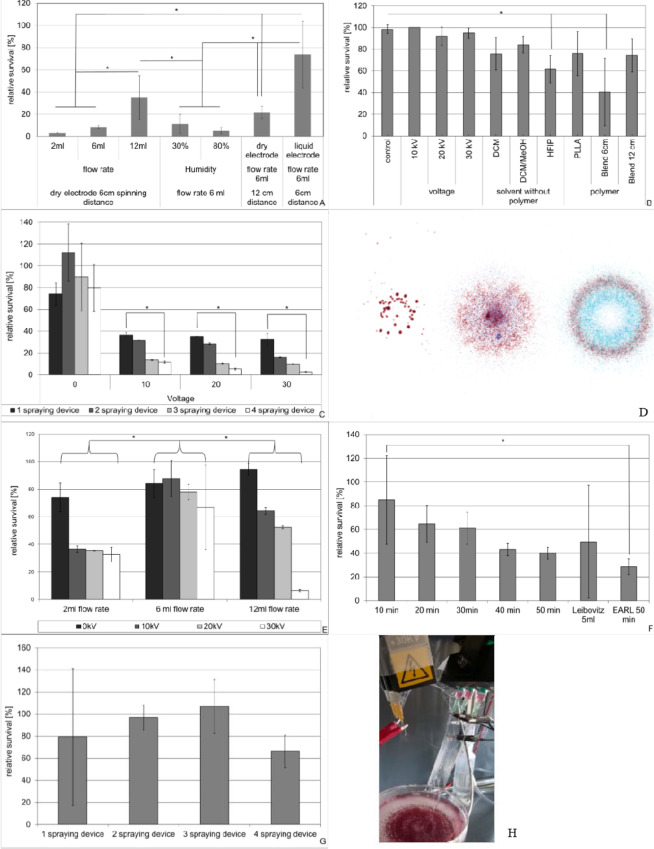
Parameters influencing cell survival during construction of cell seeded nanofiber scaffolds. Cell suspension in DMEM varying from 100000 to 400000 cells were electro sprayed from one to four devices and Scaffolds were built from 2 devices. Flow rate, voltage, solvent, scaffold polymer spinning/spraying distance and time as well as counter electrode were varied as indicated in the text. Between 6 and 27 probes of each condition were analyzed by FDA/EtBr (**B**) staining directly after scaffold production or at least 3 obtained scaffolds were analyzed by MTT assay after a culture period of 24 h (A,C,E,F,G). Jet-jet interaction between multiple spraying devices visualized by spraying of different colored inks from 1 to 3 devices (**D**). Failed deposition of nanofibers due to inappropriate spacing of the spindles (**H**). Data represent mean and standard deviation.

As shown in Fig. 3A initial cell survival rates were disappointingly low but increased while increasing the flow rate from 2 over 6 to 12 ml/h to 3% over 8% to 35% respectively. Despite the significant increase in cell survival at 12 ml/h flow rate (p < 0.001 vs. 2 ml/h; p = 0.001 vs. 6 ml/h), 35% survival was still deemed insufficient. Variation from 30% to 80% in air humidity resulted in no significant difference in cell survival. But increasing the spinning distance of the fibers up to 12 cm showed a significant positive effect on cell survival (p < 0.001 vs. 2 ml/h). Best effect on cell survival was found in the variation of the counter electrode. When exchanging the dry aluminum electrode for DMEM in a petri disc serving as a liquid counter electrode an increase up to 74% relative cell survival was found at a spraying rate of 6 ml/h at a spinning distance of 6 cm (p < 0.001 vs. 2 ml/h).

In order to assess the influence of Voltage on cell survival we electro sprayed cells suspended in DMEM from one device with a flow rate of 6 ml/h while the voltage was varied between 0–30 kV. No effect of voltage was found with cell survival rates varying between 92 and 100%. When adding spinning devices which where only loaded with different solvents but not with polymer while spraying cells a decrease in cell survival was found. This effect was statistically significant when using Hexafluorisopropanol (HFIP) as a solvent and compared to all voltage settings (p < 0.01). When PLLA polymer was added to the spinning devices, results were similar to those which used only the solvent (DCM or DCM/MeOH). When using a PLLA Col I Blend polymer in HFIP the toxic effect of HFIP was enhanced resulting in only 41% cell survival representing a statistically significant decrease when compared to the control group and to all voltage settings (p < 0.01). This effect of HFIP could be reduced when increasing the spinning distance up to 12 cm resulting in 74% cell survival (Fig. 3B).

When cells were incorporated into PLLA Nanofiber scaffolds (1–4 cell spraying devices in DMEM, flow rate 2 ml/h, 0–30 kV, 6 cm spraying distance; 2 PLLA Nanofiber spinning devices, flow rate 0.5 ml/h, 25 kV, 6 cm spinning distance; dry counter electrode) we found a strong negative correlation between cell survival and applied voltage as well as the number of spraying devices. Statistically significant lower cell survival rates were found when comparing 1 to 4 spraying devices at 10 kV, 20 kV or 30 kV (p = 0.002) (Fig. 3C).

However, this effect was not due to cell death but due to loss of cells during the spinning process as we found low cell retrieval rates within the scaffold in total cell count analysis. We attributed this to possible jet-jet interaction between multiple spraying devices once voltage was applied, which could be visualized when by spraying different colored inks from 1 to 3 devices within the same settings (Fig. 3D).

Due to the fact that flow rate had a protective effect on cell survival using dry counter electrodes we next analyzed the effect of different flow rates, using DMEM as a liquid counter electrode. No differences regarding cell survival were seen as long as no voltage was applied (p = 0.08). Once voltage was applied a flow rate of 6 ml/h revealed best results with statistically significant lower cell survival rates at 2 or 12 ml/h at 10 to 30 kV (p < 0.001 vs. 2 ml/h; p = 0.027 vs. 12 ml/h) (Fig. 3E).

Another method to enhance the number of cells within the scaffold apart from increasing the flow rate is the prolongation of spinning time while keeping flow rates low. However as shown in Fig. 3F this resulted in a time dependent but not statistically significant decrease down to 40% in cell survival rates. This decrease was independent of the medium used (DMEM, Leibovitz’s L-15 Medium, Medium 199, Earle’s Salts, Thermo Fischer scientific Waltham, MA USA) (Fig. 3F).

Another attempt to incorporate more living cells into the scaffold was the reduction of spraying distance based on the results above. But while reducing the spraying distance to 4 cm allowed for the introduction of more spraying devices without a significant decrease in relative cell survival between the number of devices (Fig. 3G), it resulted in failed scaffold deposition as the spinning distance had to be kept at 6 or 12 cm to circumvent the toxic effect of the solvent. In this setup the majority of scaffold polymer was found on the spraying needles (Fig. 3H).

Based on the iterations of spinning and spraying conditions we used the following set up for all following experiments:

A set up containing 2 spinning devices building the scaffold and 1 spraying device applying the cells was used. Spinning and spraying distance was set at 6 cm. Flow rates were set at 6 ml/h for spraying and 0.5 ml/h for spinning. DMEM was used as a liquid counter electrode. DCM/MeOH was used as solvent for PLLA scaffolds while HFIP was used for PLLA Col I Blend scaffolds.

Scaffolds thus obtained were either cultivated *in vitro* using growth or osteoinductive conditions or tested *in vivo* by implantation of the scaffold into a critical size defect of the rat scull.

### *In vitro* and *in vivo* experiments

#### PLLA scaffold

**Figure 4:**
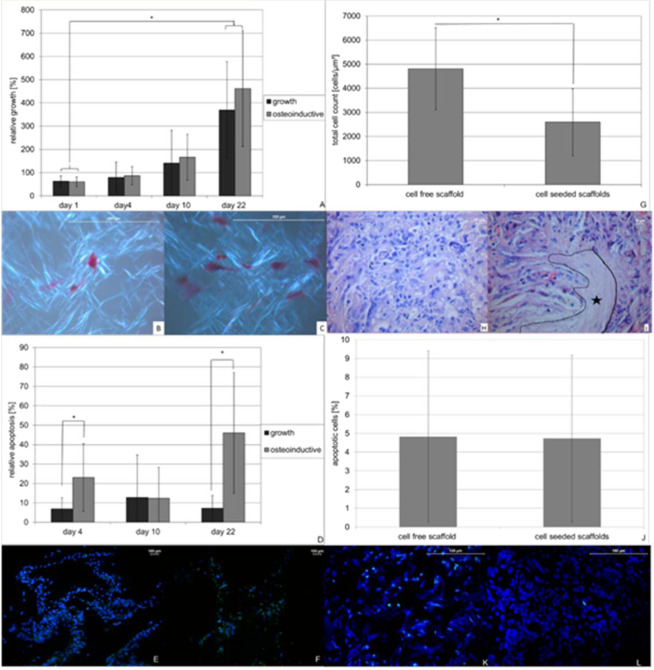
Growth and survival of cells incorporated into PLLA nanofiber scaffolds *in vitro* (**A–F**) and *in vivo* (**G–L**) using a combined process of electro spinning and electro spraying. About 200000–400000 cell were incorporated into PLLA nanofiber scaffolds and cultured under growth (3 scaffolds) and osteoinductive (3 scaffolds) conditions in DMEM medium over a period of 22 days and analyzed for metabolic activity using MTT assay in triplicate (a). Cell density/distribution was analyzed using 5 eosin stained areas of interest, prepared from 3 paraffin embedded scaffolds from each condition and time point (**B** growth-; **C** osteoinducitve conditions). Apoptosis was determined using fluorescence TUNEL assay in at least 3 histological slices of 3 paraffin embedded scaffolds of each time point and condition. (**D–F**). *In vivo* analysis was performed on 48 slices obtained from 16 defects (cell seeded group) or 30 slices obtained from 10 defects (control group). Cell densities within the scaffolds were determined by total cell count (**G**) in HE stained paraffin slices (**H,I**). Cell free area is annotated. Apoptosis was determined using fluorescence TUNEL assay in paraffin embedded histological slices by performing total and positive cell count (**J–L**). Data represent mean and standard deviation of the corresponding AOI’s.

As shown in Fig. 4A cells grew constantly over a period of 22 days. No significant differences were seen between osteoinductive or growth condition. However histological evaluation still showed cell free areas within the scaffold after 22 days of culture under both conditions (Fig. 4B,C).

TUNEL assays were performed to clarify whether apoptosis might be responsible for the cell free areas within the scaffold. As shown in Fig. 4D–F, apoptosis rates varied over time between 6 and 46%. Statistically significant differences between growth and osteoinductive conditions were seen on day 4 (p = 0.006) and day 22 (p < 0.001) with higher apoptosis rates under osteoinductive conditions.

After implantation of the scaffolds into a critical size defect of the rat skull, animals were sacrificed after 4 weeks and scaffolds were explanted. Evaluation of scaffold viability (Fig. 4G–L) still showed cell free areas within the scaffolds (Fig. 4I). When comparing cell density *in vivo* between the cell-free and the cell-seeded scaffolds after 4 weeks, overall cell density was significantly lower in the cell-seeded scaffolds (p < 0.001) (Fig. 4G). The rate of apoptosis *in vivo* was comparable between both scaffolds at approx. 5% in both scaffolds (p = 0.96) (Fig. 4J–L).

**Figure 5:**
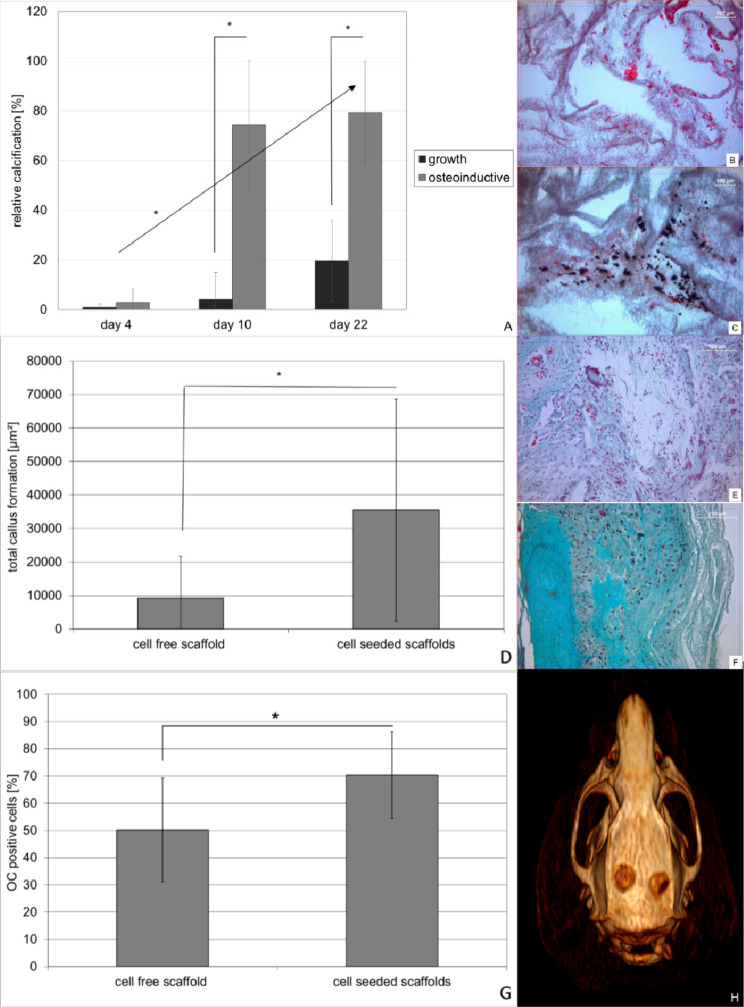
Osteoinductive potential of cells seeded PLLA nanofiber scaffolds *in vitro* (**A–C**) and *in vivo* (**D–G**). PLLA nanofiber scaffolds containing 400000 cells were cultured under growth and osteoinductive conditions in DMEM medium over a period of 22 days (3 scaffolds per time point and condition). Osteoinductive potential *in vitro* was determined on 5 areas of interest using von Kossa staining of paraffin embedded histological slices (**A**, **B** growth- **C** osteoinductive conditions). *In vivo* bone formation was determined on 48 areas of interest obtained from 16 defects (cell seeded group) or 30 areas of interest form 10 defects (control group) either by masson goldner staining (**D**) of cell free (**E**) or cell containing (**F**) scaffolds as well was immunohistochemically staining of OC (**G**). Data represent mean and standard deviation of the corresponding AOI’s.

In order to evaluate osteoblast differentiation and bone formation *in vitro* (Fig. 5A–C) and *in vivo* (Fig. 5D–G), scaffolds were stained von Kossa after 4, 10 and 22 days of cultivation. Under osteoinductive conditions calcified area was 74% and 79% after 10 and 22 days of culture respectively representing a significant difference compared to the growth conditions (p < 0.001 on day 10; p = 0.006 on day 22). Additionally the increase in calcified area under osteoinductive conditions from day 4 to day 10 and day 22 respectively also yielded statistical significance (p < 0.001 each) (Fig. 5A–C).

*In vivo* cell-seeded scaffolds showed a total callus formation of approximately 35000 µm², which was at least 18% of the scaffold, while cell-free scaffolds only showed a total callus formation of 9200 µm², representing 5% of the scaffold and a statistically significant difference (p < 0.001) (Fig. 5D–F). This finding was supported by immunhistochemical staining for osteocalcin showing 70% positive cells within the cell-seeded scaffold compared to 50% in the cell-free scaffold representing a significant difference (p < 0.001) (Fig. 5G).

However radiologic examination by means of a CT scan showed no differences in mean Hounsfield units (HU) within the former defects (cell-free scaffold 305 ± 119 HU vs. cell-seeded scaffolds 266 ± 230 HU) indicating that the hard callus formation was not affected by cell incorporation after 4 weeks.

#### PLLA-collagen blend scaffold (PLLA Col I Blend)

**Figure 6:**
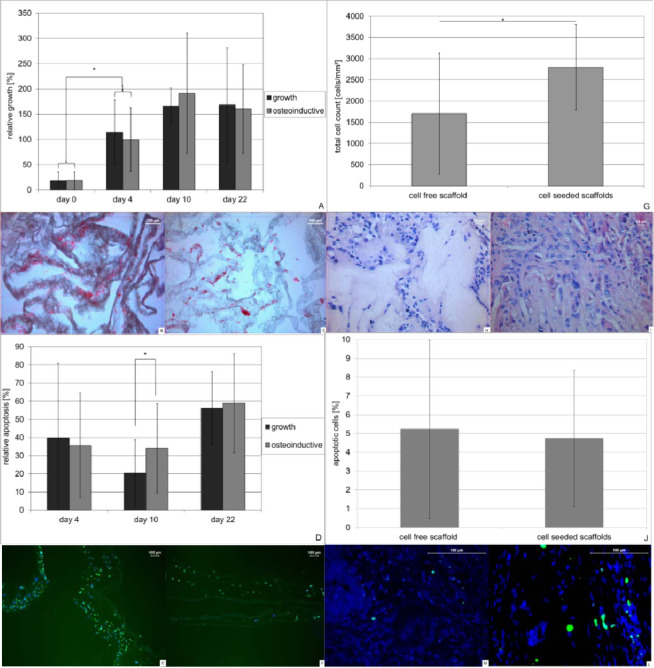
Growth and survival of cells incorporated into PLLA-collagen blend nanofiber scaffolds *in vitro* (**A–F**) and *in vivo* (**G–L**) using a combined process of electro spinning and electro spraying. About 700000–2000000 cell were incorporated into PLLA nanofiber scaffolds and cultured under growth (3 scaffolds) and osteoinductive (3 scaffolds) conditions in DMEM medium over a period of 22 days and analyzed for metabolic activity using MTT assay (a). Cell density/distribution was analyzed using 5 eosin stained areas of interest, prepared from 3 paraffin embedded scaffolds from each condition and time point (**B** growth-; **C** osteoinducitve conditions). Apoptosis was determined using fluorescence TUNEL assay in at least 3 histological slices of 3 paraffin embedded scaffolds of each time point and condition. (**D–F**). *In vivo* analysis was performed on 48 slices obtained from 16 defects (cell seeded group) or 30 slices obtained from 10 defects (control group). Cell densities within the scaffolds were determined by total cell count (**G**) in HE stained paraffin slices (**H,I**) Apoptosis was determined using fluorescence TUNEL assay in paraffin embedded histological slices by performing total and positive cell count. (**J–L**). Data represent mean and standard deviation of the corresponding AOI’s.

As shown in Fig. 6A cells grew constantly over a period of 10 days. From day 10 to day 22 of culture the cell densities remained constant. No significant differences were seen between osteoinductive or growth condition. However histological evaluation still showed cell free areas within the scaffold after 22 days of culture under both conditions (Fig. 6B,C).

TUNEL assays were performed to clarify whether apoptosis might be responsible for the cell free areas within the scaffold. As shown in Fig. 6D–F, apoptosis rates varied over time between 18 and 59% with no statistical differences on day 4 (p = 0.46) and day 22 (p = 0.63) but a significant difference on day 10 (p = 0.017) between growth and osteoinductive conditions.

Four weeks after implantation of the scaffolds into a critical size defect of the rat skull animals were sacrificed and scaffolds explanted. Evaluation of scaffold viability (Fig. 6G–L) still showed cell free areas within the scaffolds (Fig. 6H). But when comparing cell density *in vivo* between the cell-free and the cell-seeded scaffolds, the cell-free scaffolds showed a much higher amount of cell free areas after 4 weeks (Fig. 6H,I) and overall cell density was significantly higher in the cell-seeded scaffolds (p < 0.001) (Fig. 6G). The rate of apoptosis *in vivo* was comparable at approx. 5% in both scaffolds (p = 0.99) (Fig. 6J–L).

**Figure 7:**
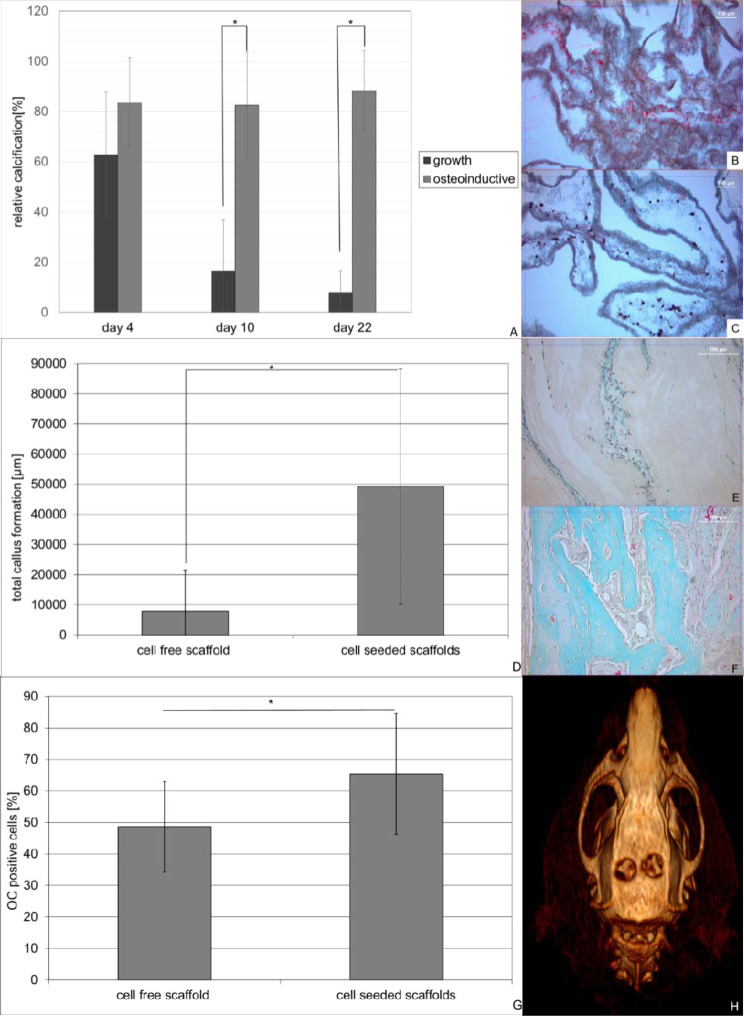
Osteoinductive potential of cells seeded PLLA -collagen blend nanofiber scaffolds *in vitro* (**A–C**) and *in vivo* (**D–G**). PLLA nanofiber scaffolds containing 400000 cells were cultured under growth and osteoinductive conditions in DMEM medium over a period of 22 days (3 scaffolds per time point and condition). Osteoinductive potential *in vitro* was determined on 5 areas of interest using von Kossa staining of paraffin embedded histological slices (**A**,**B** growth- C osteoinductive conditions). *In vivo* bone formation was determined on 48 areas of interest obtained from 16 defects (cell seeded group) or 30 areas of interest form 10 defects (control group) either by masson goldner staining (**D**) of cell free (**E**) or cell containing (**F**) scaffolds as well was immunohistochemically staining of OC (**G**). Data represent mean and standard deviation of the corresponding AOI’s.

In order to evaluate osteoblast differentiation and bone formation *in vitro* (Fig. 7A–C) and *in vivo* (Fig. 7D–G), scaffolds were stained von Kossa after 4, 10 and 22 days of cultivation. Under osteoinductive conditions calcified area was 83% and 88% after 10 and 22 days of culture respectively representing a significant difference compared to the growth conditions (p < 0.001 on day 10; p < 0.001 on day 22). The increase in calcified area under osteoinductive conditions from day 4 to day 10 and day 22 respectively was not statistically significant (Fig. 7A–C). *In vivo* cell-seeded scaffolds showed a total callus formation of approximately 50000 µm², which was at least 22% of the scaffold, while cell-free scaffolds only showed a total callus formation of 7900 µm², representing 4% of the scaffold and a statistically significant difference (p < 0.001) (Fig. 7D–F). This finding was supported by immunhistochemical staining for osteocalcin showing 65% positive cells within the cell-seeded scaffold compared to 49% in the cell-free scaffold representing a significant difference (p < 0.001) (Fig. 7G).

CT scans of the skull confirmed the histological results showing significant differences in mean Hounsfield units (HU) within the former defects (cell-free scaffold 113 ± 51 HU vs. cell-seeded scaffolds 317 ± 227 HU).

## Discussion

The colonization of nanofiber scaffolds *in vivo* has been shown to be a critical factor in the setting of successful bone tissue engineering^42^. In case of the PLLA Col I Blend, this is mainly limited by the pore size of nanofiber scaffold^43,44^.

With regard to tissue engineering the direct incorporation of cells into the nanofiber scaffold could circumvent the time-consuming colonization process and the limiting factor of pore sizes. Here, a combined process of multijet electro spraying of cells and electrospinning of nanofiber scaffolds is a promising approach.

A prerequisite regarding this approach is that the cells survive the electro spraying process in large numbers. And indeed several authors showed that different cell types survive the electro spraying process: smooth muscle cells (Stankus *et al*.^51^, Stankus *et al*.^48^), human astrocytoma cells (Jayasinghe and Townsend-Nicholson^52^) or osteoblast cell line MG63 (Paletta *et al*.^49^). In this study we describe a multi-jet electro spraying and -spinning procedure of mesenchymal stem cells incorporated in a PLLA or PLLA-Col I Blend scaffold, identify parameters which influence cellular survival and we characterize the osteoinductive potential of scaffolds thus obtained *in vitro* and *in vivo*.

Among the factors investigated, dehydration of the cells after spraying was a major limiting factor. This could effectively be counteracted by introducing a wet counter-electrode, leading to sufficient cellular survival. Other attempts like increasing general humidity or increasing flow rates were not or less effective when performed with a dry counter electrode.

These findings are in line with findings of Braghirolli *et al*.^53^, who reported more than 90% survival when only electro spraying mesenchymal stem cells (MSCs), indicating that voltage has no influence on cell survival. However, voltage has some influence within this system, namely on cell retrieval, especially when a multi-jet system is used. The decrease in cell retrieval within our multi-jet system was not due to cell death but due to loss of cells during the spinning/spraying process. We attribute this to jet-jet interaction between multiple spraying and spinning devices once voltage is applied. Theron *et al*.^54^ analyzed the interaction of multiple jets in electrospinning and found that the jets are pushed away from their neighbors by the coulombic forces caused by the latter. This resulted in deposition of material outside of the collecting area/counter electrode. In our opinion this effect is responsible for the reduced cell retrieval as well as the reduction in scaffold mass during electrospinning.

While we found no negative impact of the applied voltage on cell survival, we did find a negative impact of the polymer solvent on cell survival. Although this effect could be minimized by increasing the spinning distance and thus increasing solvent evaporation, a compromise is necessary because a set up using optimal distances for both electrospinning and -spraying resulted in failed deposition of the scaffold. The negative impact of the polymer solvent was most prominent when HFIP was used. This might be attributed to the water solubility of HFIP, possibly leading to local inhibitory concentrations of HFIP by mixing of HFIP with the DMEM of the liquid counter electrode.

Data on mechanical stability is, in summary, not in an area which would represent clinically significant stability when putting the results into a context of primary stable bone replacement. Still, scaffolds are mechanically stable to be handled during surgery without problems. Whether changes in mechanical properties between scaffolds result in a biomechanical difference or differences in cell proliferation and differentiation is currently unclear. Clear, in contrast, is the relationship between the ability of the scaffold to promote cellular ingrowth or colonization and fiber diameter/pore size of the scaffold. Szentivanyi *et al*.^44^ showed in their review, that cellular migration into nanofiber scaffolds is facilitated within an optimal corridor of 5–50 µm pore size and a fiber diameter above 250 nm. In the present study this corridor was necessarily circumvented by the co-axial spinning and spraying process as pore size differed between 0.5 µm and 2.9 µm and fiber diameter differed between 186 nm and 557 nm. Similar pore sizes and fiber diameter for PLLA-Col I Blend scaffolds were found by Schofer *et al*.^43^ as well as showing the failed scaffold colonization of these scaffolds *in vivo* by Schofer *et al*.^42^.

When comparing both scaffolds significantly lower cellular densities as well as lower early metabolic activity could be perceived within the PLLA-Col I Blend.

These results are contradictory to e.g. the results of Schofer *et al*.^36^ who showed increased growth rates when colonizing PLLA-Col I Blend scaffolds with human MSCs in comparison to PLLA scaffolds. The underlying principle is probably the effect of biologically active peptide sequences on e.g. collagen or gelatin and their integrine mediated cellular recognition^55,56^. As the structural changes of collagen caused by electrospraying^57^ do not limit it’s biological effects on promoting cellular proliferation as shown by^58^, other factors related to the used celllines as well as growth conditions seem more likely to affect the contradictory decrease in metabolic activity and cellular densities within the PLLA-Col I Blend scaffolds.

When taking the higher apoptosis rates within the PLLA-Col I Blend scaffolds during *in vitro* cultivation into account (Fig. 4D vs. 6D), the most likely reason is the lingering toxic effect of HFIP due to its water-solubility.

Using PLLA and a PLLA-Col I Blend as polymers resulted in cell survival, proliferation and differentiation over a period of 22 days of MSCs in the present study. Similar results for proliferation but without evaluating differentiation after incorporation in a PLGA scaffold were found by Braghirolli *et al*.^53^. A different approach evaluated by Zanatta *et al*.^50^ was a monoaxial spinning and spraying process using a water-soluble polymer (PVA – polyvinyl alcohol) and MSCs or Mononuclear cells (MNCs). This approach resulted in unsatisfactory cellular survival of 19,6% using MSCs and 8,38% using MNCs, thus indicating that the separation of polymer and cells in a coaxial process is the most promising approach.

In bone tissue engineering extracellular matrix formation is, besides cell survival and proliferation, of key importance. The capacity of nanofiber scaffolds to allow and promote cellular differentiation towards osteoblasts was shown when hMSCs were cultured on different scaffold-types (PLLA, Collagen, PLLA-Collagen Blend)^36,59^. In the present study, evidence for osteoblastic differentiation after electro spraying of MSCs was found by expression of OC and positive von Kossa staining, indicating that both nanofiber-scaffolds used (PLLA and PLLA-Col I Blend) facilitate bone formation after the coaxial electro spraying and –spinning process. This indicates, that the electro spraying process does not compromise the osteogenic potential of MSCs, which is in line with the results of Braghirolli *et al*.^53^.

When comparing both scaffolds regarding the von Kossa staining, a markedly higher calcium integration could be found within the PLLA-Col I Blend (Figs. 5A vs. 7A). Thus implying that the integrin mediated osteoinductive effect of collagen as described by Mizuno and Kuboki;^60^ Salasznyk *et al*.^61^; Xiao *et al*.^62^ and observed by Schofer *et al*.^36^ is maintained within the PLLA-Col I Blend scaffolds.

The consequent transfer of these *in vitro* results into *in vivo* experiments is lacking in current literature. Direct comparison to other nanofiber based cell-containing scaffolds is difficult because to the best of our knowledge this work is currently the first to present *in vivo* results of the described co-axial method for direct incorporation of cells into a nanofiber scaffold. But the *in vivo* results confirmed our *in vitro* results with respect to proliferation and differentiation. Additionally, a significantly better callus formation and higher percentage of healed bone was found when compared to the former cell-free scaffolds^42,63^. Although the period of four weeks represents an early phase of defect healing, the results show consistently better defect healing not only by radiological but also by histological evaluation compared to the former cell-free scaffolds^42,63^.

A possible clinical application could be the treatment of large bony defects in bone tumor surgery as well as in revision arthroplasty or possibly in pseudarthrosis. But as in autologous chondrozyte transplantation, some amount of preparation time will be required. A bone marrow aspirate would be sufficient to expand MSCs and create a scaffold of appropriate size. Whether it also makes sense to differentiate the scaffolds into finished bone *in vitro* before implantation remains to be determined.

To evaluate the impact of the presented co-axial procedure, 3D bio-printing represents a comparable approach. Although to the best of our knowledge no *in vivo* results for bone regeneration with printed cell-containing scaffolds are currently available, *in vitro* data is comparable. With respect to cell survival or osteogenic differentiation Levato *et al*.^64^ bio printed MSCs and found a viability of approximately 90% which is comparable to the cell survival obtained by electro spraying from DCM/MeOH. Koch *et al*.^65^ could show no significant increase of apoptosis or DNA fragmentation using the laser assisted bio printing method (LIFT) and hMSCs. Osteogenic differentiation could also be maintained as shown by Gruene *et al*.^66^ using LIFT and mesenchymal stem cells. The one advantage of bio-printing over the presented co-axial remains the ability to control local cellular deposition.

In conclusion, the presented data shows that multipotent bone derived mesenchymal cells can be electro spun into a nanofiber scaffolds. The resulting scaffolds are vital *in vitro* and show osteoblastic differentiation. *In vivo* they lead to the healing of critical bone defects. Regarding the PLLA-Collagen type I scaffolds, the cell based approach seems to be superior to unpopulated nanofiber constructs. Clinical studies must show whether this advantage justifies a two-stage approach.

## Materials and Methods

### Construction of cell-containing nanofibre scaffolds

In order to incorporate cells into nanofiber scaffolds, a parallel approach combining electrospinning of polymer and electro spraying of cells – based on Paletta *et al*.^49^ was used. Therefore an electrospinning apparatus was constructed with two independent controlled spin heads. Each can be loaded with up to 4 syringes with 1, 2 or 5 ml volume. Flow rates of each spin head can be adjusted from 0 ml/h to 12 ml/h. Each spin head is connected to a separate power supply. The voltage applied to each spin head can be regulated between 0–30 kV. The voltage applied to the counter electrode can be set between 0 and −30 kV in case of static dry counter electrodes. When a rotating counter electrode was used the voltage was limited to −5 kV. Rotation can be set between 0 and 60 rpm. When liquid counter electrodes were used, petri-discs containing medium were connected to the electrode. The spinning distance can be varied between 4 and 30 cm.

With respect to safety the apparatus is set in a 100*120*120 cm cabinet with automatic closing doors opening only after high-voltage shut-down. Solvent vapor can be withdrawn by suction using an integrated ventilator, connected to a filter-system (Fig. 1).

### Polymer preparation

For preparation of poly-(l-lactic acid) (PLLA) nanofibres by electrospinning, a 4% (w/w) PLLA solution (Resomer L210, Boehringer, Ingelheim, Germany) was prepared by solving the PLLA in dichloromethane (DCM) or dichloromethane/methanol (DCM/MeOH)^36,49^. In order to functionalize PLLA with type I collagen, PLLA and type I collagen were dissolved in hexafluoroisopropanol (HFIP) in the desired ratio of 4:1 PLLA:collagen until a 4.5% (w/v) polymer solution was obtained^59,67^.

### Isolation of multipotent bone marrow derived mesenchymal cells (MSCs)

Multipotent bone derived mesenchymal cells (MSCs) were isolated from rat femur according to de Hemptinne *et al*.^68^ and cultured in growth medium (DMEM supplemented with 10% fetal bovine serum and 5 mL of streptomycin/penicillin solution (Biochrom Berlin Germany) at 37 °C in a humidified atmosphere containing 5% CO2. For experiments aliquots of cells were immunostained for CD 90 and CD 105 (antibodies by Santa Cruz Biotechnology, Inc. Dallas, Texas, USA). Passages containing more than 80% positive cells were used.

### *In vitro* assessment

Vital staining was performed with 30 μg/mL phosphate buffered fluorescein diacetate (FDA) (Sigma-Aldrich, Munich, Germany) and 38 μg/mL ethidium bromide (Sigma-Aldrich, Munich, Germany) in phosphate buffered saline (PBS). Relative survival was determined by counting positive (green) and negative (red) cells. Metabolic activity was assessed with 3-(4,5-dimethylthiazol-2-yl)−2,5-diphenyltetrazolium bromide (MTT) using a commercially available kit (R&D Systems, Minneapolis, USA) according to the manufacturer’s instruction. Relative survival was calculated from corresponding calibration curves.

For light microscopy analysis, cell scaffolds were fixed with 3,5–3,7% formalin dehydrated in serial solutions of ethanol (70–100% for 10 min each) and paraffin embedded. Sections of 5 μm thickness were stained with a Masson Goldner Kit (Merck, Darmstadt, Germany) according to the manufacturer’s instruction. Van Kossa staining was performed using 1% aqueous silver nitrate solution for 1 h followed by several washing steps in distilled water and incubation in 5% sodium thiosulphate for 5 minutes.

Apoptosis was determined using the “*In Situ* Cell Death Detection Kit, Fluorescein” (Roche (Merck, Darmstadt, Germany)) following the instructions of the manufacturer. Samples were analyzed directly or embedded with antifade using a fluorescence microscope (Leica DM5000 (Wetzlar, Germany)) with an excitation wavelength in the range of 450–500 nm (e.g., 488 nm) and detection in the range of 515–565 nm (green). It should be noted that in some cases there may be an unspecific background staining.

For immunohistological staining the slices were rehydrated and endogenous peroxidase activity was quenched with a Bloxall solution (Vector laboratories, Burlingame, CA USA), blocked with goat or horse serum (Vector laboratories, Burlingame, CA USA) and incubated overnight at 4–8 °C with a polyclonal IgG antibody against osteocalcin (Santa Cruz Biotechnology, Inc. Dallas, Texas, USA), diluted 1:100.

Slices were then incubated with a biotinylated secondary antibody (Vector laboratories, Burlingame, CA USA) for 30 minutes at room temperature and incubated for 60 minutes (Elite Vectastain ABC kit, Vector laboratories, Burlingame, CA USA) at room temperature. Finally, the slices were visualized with diaminobenzidine (DAB) (Dako Carpinteria, CA USA) and counterstained with Gill’s hematoxylin solution (Merk, Darmstadt, Germany) for 20 seconds. Negative controls, created by incubated without primary antibody, were treated in the same manner. A Leica DM5000 (Wetzlar, Germany) was used for microscopy.

#### Mechanical testing

Stability of the scaffold was determined using a uniaxial servohydraulic testing machine Instron® 5566 (Instron Cor., Darmstadt, Germany) with a custom-made holding device. Axial load was applied and load to failure analysis was done at a crosshead speed of 10 mm/min without any pretension or preconditioning. The data were collected at 100 ms intervals using the instrument-specific Blue Hill® Software (Version 2.17, Instron Cor., Darmstadt, Germany) and maximum load and elongation at break were calculated.

#### Scanning electron microscopy

For scanning electron microscopy (SEM) of electrospun fiber mats air dried specimens were sputter-coated with platinium (Pt) under vacuum and examined on a scanning electron microscope (SEM) model JSM-5410 (JEOL, Japan) at an accelerating voltage of 2.00 kV. Based on porosity and fiber diameter pore size was calculated based on Tomadakis and Robertson^69^.

### *In vivo* assessment

Adult male Sprague-Dawley rats (Charles River Laboratories, Sulzfeld, Germany) were used in the experiment. Animals were kept in plastic cages (Macrolon Type III) with a maximum of two animals per cage in a room maintained at a constant temperature of 22 °C, with a 12 h light/dark cycle. Animals had free access to drinking water and standard laboratory food pellets. All experiments were carried out in strict accordance with the recommendations in the Guide for the Care and Use of Laboratory Animals of the NIH and approved by the local Animal Ethics Committee Regierungspräsidium Giessen (reference number V 54–19 c 20 15 h 01 MR 20/21 Nr. 38/2015).

Under the principles of the 3 R’s (Russel and Burch) specimen from our previous work using cell free scaffolds were reevaluated under the same conditions as stated below to serve as comparison for the present study using cell-seeded scaffolds^43,63^.

#### Surgery

Animals were divided into two groups (1: cell seeded PLLA nanofiber scaffolds; 2: cell seeded PLLA-collagen type-I nanofiber scaffolds). Power analysis based on alpha = 0.05 and beta = 0.20 and a relevant difference of 15% defect area resulted in eight animals per group. Bilateral full thickness critical size calvarial defects were created, sparing the sagittal sinus. Both defects were randomly filled with one of the above mentioned scaffolds. Surgery was performed under general anesthesia by weight adjusted intraperitoneal injection of xylazine 2% (RompunH, 5 mg/kg body weight, Bayer Animal Health, Leverkusen, Germany) and ketamine hydrochloride (Ketamin WDT, 100 mg/kg body weight, WDT, Garbsen, Germany). To prevent wound infection each rat received a subcutaneous injection of 50 mg/kg body weight Ampicillin directly after anesthesia. Under aseptic conditions a sagittal incision over the skull was made and, after preparation including the periosteum, the bone was exposed. Two bilateral 5 mm full thickness critical size defects (CSD) were created using a trephine bur. Constant irrigation with sterile physiological saline solution was applied to prevent heat accumulation. After implantation of the cell-seeded scaffold according to group, the site was closed by suturing the overlying tissue and skin by absorbable sutures. Animals were sacrificed after 4 weeks by CO_2_-asphyxiation while being under anesthesia as described above.

After animals were sacrificed radiographic evaluation was performed using computed tomography (CT) imaging (Somatom Definition, Siemens Medical Systems, Erlangen, Germany) with a resolution of 0.3 mm. Images were transferred to an image analysis workstation using a picture archiving and communication system (PACS) for evaluation. In order to analyze bone tissue repair, the radiological density was measured by placing a region of interest (ROI) over the former defect side. Bone density was measured in Hounsfield units (HU).

The defect sites were removed together with a small amount of surrounding bone and soft connective tissue. These samples were immediately fixed in 4% buffered formalin for three days and then decalcified in EDTA-solution (OsteosoftH, Merck, Darmstadt, Germany) over a period of 18 days. The bone specimens were then dehydrated in graded alcohol solution and cedar wood oil and finally embedded in paraffin. Five µm slices were cut on a rotation microtome (RM2055, Leica Microsystems, Bensheim, Germany).

Basic histological assessment was done using standard Hematoxylin-Eosin staining. Histomorphometric analysis was performed in three Masson-Goldner trichrome-stained sections at a primary magnification of 20-fold using a digital microscope (DM5000, Leica Microsystems, Bensheim, Germany) and QUIPS with Qwin analysis software (Leica Microsystems). Formation of new bone was calculated in relation to the defect area of each section and expressed as a percentage. Immunohistological evaluation was carried out by selecting five representative regions of interest (ROI) at 63-fold magnification connecting both defect margins together. Open Source software Image J was used for cell counting.

### Statistical analysis

All *in vitro* results are given as mean ± standard deviation (SD) of at least 3 different independent assays. All *in vivo* results are given as mean ± SD. Exact data is given on each graphic. Eight animals representing 16 defects per group were used according to the a-priori power analysis setting β = 0,8. Control group animal-data from prior work consisted of 10 defects. Before statistical testing, data was checked for normal distribution using the Shapiro-Wilk test. In case of a normal distribution, results were compared using ANOVA and post-hoc LSD test or directly by Student’s t-test in case of 2 groups. In case of a non-normal distribution, non-parametric testing was performed using Kruskal-Wallis test and post-hoc Mann-Whitney-U-test. A p-value of less than 0.05 was considered statistically significant after Bonferroni-adjusting for multiple testing. Calculations were performed using SPSS^®^ (IBM^®^, Build 1.0.0.1327).
